# A targeted strategic peer support intervention to increase adherence to video teletherapy exposure and response prevention treatment for obsessive-compulsive disorder: a retrospective observational analysis

**DOI:** 10.3389/fnhum.2023.1251194

**Published:** 2023-10-30

**Authors:** Christopher E. Murphy, Andreas Rhode, Jeremy Kreyling, Scott Appel, Jonathan Heintz, Kerry Osborn, Kyle Lucas, Reza Mohideen, Larry Trusky, Stephen Smith, Jamie D. Feusner

**Affiliations:** ^1^Michener Institute of Education at the University Health Network, Toronto, ON, Canada; ^2^NOCD Inc., Chicago, IL, United States; ^3^Biostatistics Analysis Center, Perelman School of Medicine, University of Pennsylvania, Philadelphia, PA, United States; ^4^Department of Psychiatry, University of Toronto, Toronto, ON, Canada; ^5^General Adult Psychiatry & Health Systems Division, Centre for Addiction and Mental Health, Toronto, ON, Canada; ^6^Department of Women’s and Children’s Health, Karolinska Institute, Stockholm, Sweden

**Keywords:** peer support, OCD, adherence, telehealth, machine learning, cognitive-behavioral therapy, exposure and response prevention

## Abstract

Exposure and response prevention (ERP) therapy, a form of cognitive-behavioral therapy, is a first-line, evidence-based treatment for obsessive-compulsive disorder (OCD) for adults and children. It is effective for the majority of those who engage in it, but treatment adherence can be challenging for some due to the stress involved in the treatment as well as different life circumstances that arise. To help improve treatment adherence, NOCD, a provider of video teletherapy ERP, identifies those at risk of non-adherence using a prediction algorithm trained on a data set of *N* = 13,809 and provides targeted peer support interventions by individuals (“Member Advocates”) who successfully completed ERP treatment for OCD. Member Advocates, using lived OCD experience as well as experience with ERP, engage at-risk patients through digital messaging to engage, educate, and encourage patients in the early stages of treatment. From June 2022 to August 2022, *N* = 815 patients deemed at risk were reached out to and *n* = 251 responded and engaged with the Member Advocates. In the at-risk patients who engaged, the intervention resulted in a significant mean 30.4% more therapy hours completed compared to those who did not engage. Additionally, engaged patients had greater reductions in OCD severity. These results have implications for how data science, digital interventions, and strategic peer-to-peer communication and support can be combined to enhance the effectiveness of treatment.

## Introduction

Exposure and response prevention (ERP) therapy is a type of cognitive-behavioral therapy that expert consensus guidelines deem a first-line treatment for obsessive-compulsive disorder (OCD) in adults and children (National Collaborating Centre for Mental Health (UK), 2006 Koran et al., 2007). ERP is the most effective psychotherapeutic treatment for OCD and can result in lasting improvements (Stewart and Chambless, 2009; McKay et al., 2015; Öst et al., 2015; Skapinakis et al., 2016; Reid et al., 2021). However, it is critical that individuals adhere to their therapy regimen in order to experience these benefits. Non-adherence to OCD therapy is a common challenge in real-world clinical settings, affecting 31–65% (Bergin and Garfield, 1994; Mancebo et al., 2011). In a longitudinal observational study (*N* = 89), 31% of those who started CBT dropped out prematurely and 57% did not attend sessions at a minimum recommended frequency of once or twice weekly (Mancebo et al., 2011). In a retrospective survey, anxiety about the treatment and perceived barriers such as being too busy, financial barriers (out of pocket costs or non-coverage of treatment by their health plan) or CBT not being available were the most common self-reported reasons for not receiving or completing CBT for OCD (Mancebo et al., 2008). Possible risk factors include female sex, which has been associated with higher non-adherence for remote therapy compared with face-to-face in-person CBT for OCD (Salazar de Pablo et al., 2023). Identifying early on who is at risk for non-adherence would allow for an efficient means to provide targeted interventions to maintain adherence.

As increasingly more patient data is collected in electronic health records and other patient-related data such as on clinical apps, robust machine learning models can be trained and deployed to aid in clinical decision making. Prediction models may aid in diagnoses or prediction of patient outcomes. Precision medicine, taking advantage of “-omics” level data and machine learning, advances our ability to identify and characterize human disease in patients with Type I diabetes and Crohn’s disease, for example (MacEachern and Forkert, 2021). Yet, classification tasks for mental health disorders can be more challenging due to much of the clinical data relying on subjective responses (which may not always be accurate or reliable), comorbidities, and gaps in data (Chung and Teo, 2022). Adherence to CBT can be predicted by supervised machine learning algorithms by training models with human-labeled outcomes of real patients’ therapy courses. In this way, risk for non-adherence and individual risk factors themselves may be identified for future patients. Once identified, at-risk patients can be targeted for personalized outreach to help reduce their risk.

One method of personalized outreach is through peer support interventions. Peer support interventions have emerged as a promising way to augment care across a range of conditions (Cook et al., 2010). It involves individuals with lived experiences of a condition providing support and guidance to others currently facing similar experiences. In behavioral health, peer support interventions can promote recovery, engage patients, and improve quality of life for individuals with mental health or substance use disorders (Chinman et al., 2014). Digital peer support interventions also appear to be effective in improving the lives of those with severe mental illness (Fortuna et al., 2020). Significant improvements in depressive symptoms, hospital admissions, length of stay, and patient satisfaction have been observed for interventions consisting of peer-to-peer networks combined with evidence-based practice (Aschbrenner et al., 2016; Gucci and Marmo, 2016; Schlosser et al., 2018). Longitudinal data is needed to determine whether peer support can sustain these improved outcomes over time.

Previously, we reported outcomes from an ERP-based treatment program in adults with OCD (Feusner et al., 2022). The patients treated at that time had *ad hoc* access to peer support throughout their treatment, although there was no targeting of patients who were at higher risk of non-adherence and the effects of engaging with peer support were not measured. Given the problematic nature of OCD treatment non-adherence, and that machine learning has the potential to assist clinical management through prediction of clinical outcomes, a targeted approach to addressing non-adherence was implemented. We developed a prediction model to identify who might be most at risk of non-adherence, and therefore most in need of peer support. In the current, retrospective observational analysis, we examined if these peer support interventions that were targeted to those at elevated risk of non-adherence improved their adherence to virtual ERP compared with those who did not engage in these interventions.

## Materials and methods

### Patients

Data were analyzed from patients who were treated at NOCD. NOCD is a digital behavioral health treatment program that provides one-on-one ERP teletherapy with trained therapists to adults and children, in all 50 U.S. states and in Canada, Australia, and the U.K. In addition, NOCD provides patients with the option of peer support. This consists of interactions with paid advocates (“Member Advocates”) who have lived experience with OCD (have or have had OCD in their lifetime), were treated successfully with ERP, and can offer practical and emotional support at critical points during therapy. Patients are referred to as “members” at NOCD.

### Prediction model

We developed a model to predict which members might be adherent vs. non-adherent to ERP treatment. To achieve this, we trained and tested a logistic regression model using data from *N* = 13,809 individuals aged 4 to 88 (Supplementary Table S1) who received treatment from NOCD prior to implementing the peer advocate intervention. The prediction was specifically to identify who was likely to be adherent to therapy, defined as having completed at least 12 appointments. For training, we used features collected up to the third visit of therapy for each member. Some continuous features were converted into binary categories, with quantitative attributes being binned into value ranges (for ease of interpretation, *post hoc*).

The logistic regression model, trained on *n* = 10,322 and tested in a holdout sample of *n* = 3,487, consisted of 49 features, each with an associated weight, as well as a bias offset. These features (see Supplementary Table S2) were initially hand-selected by one of the authors (JDF) with extensive clinical experience in treatment of OCD. The feature list was then reviewed by a second experienced clinician on the clinical leadership team to obtain consensus (consensus was achieved for all features). The selected features represented a range of baseline patient demographic and psychometric factors, characteristics of their treating therapists, and patient behaviors prior to treatment and in the first 3 weeks of treatment (such as app use and messaging, e.g.) that were deemed to potentially impact their adherence. Alternative logistic regression models were iterated on the complete case analysis. A thorough model selection process was completed, including stepwise forward selection and manual selection techniques. The final model chosen was the one with the best average fit.

In the training sample the model demonstrated an AUC of 0.643 and in the holdout sample, an AUC of 0.653. The sensitivity and specificity for the trained model were 61.3 and 59.4%, respectively. Alternative models such as random forest and support vector classification were also tested but did not yield better accuracy. The alternative models were similarly allowed to select from all the available hand-selected features and were iterated through using a combination of forward stepwise selection and manual addition/removal. The final trained logistic regression model was then used to predict new members’ risk to non-adherence between June 2022 and August 2022.

### Peer support intervention

The intervention took place between June–August 2022. Six Member Advocates reached out to patients between Session 1 and Session 12. A portion of the patients identified as at-risk by the prediction model were selected by Member Advocates to contact. Due to Member Advocates’ time constraints, they selected as many as their time allowed each business day to contact, trying to prioritize patients who had the soonest upcoming therapy appointments. As a result, 815 of 1,142 high-risk individuals were contacted.

Member Advocates work with patients who are not yet in, but contemplating or pre-contemplating, therapy, as well as those who are currently active in treatment. They help build rapport, understanding, education and use their individual experiences to encourage ERP therapy. Member Advocates can address initial concerns or questions about treatment. ERP necessitates inducing distress as part of the therapeutic process; subsequently, this can cause apprehension for new patients, which Member Advocates can help them better understand. In general, a goal of Member Advocates is to provide a positive experience for patients, starting from the scheduling of their first session with an ERP therapist to later stages of their treatment. As an option, members can come to Member Advocates at any time they feel they are uncomfortable with their therapist, unhappy with their experience, or if they need peer support and encouragement to continue with ERP. Member Advocates are trained to not provide reassurance in certain cases, as reassurance-seeking is a common compulsive behavior - similar to checking if one is doing something the “right” way, for example - thus, providing reassurance ultimately makes symptoms worse. Member Advocates receive support and supervision when needed from licensed clinicians, but themselves do not provide any clinical treatment.

The interventions for high-risk patients identified with the prediction model consisted of two phases of messaging through a secure chat platform: The first phase was an initial, standardized direct message that inquired about the patient’s therapy experience at that specific point in their treatment. There were two different initial standardized messages sent based on whether the patient had further session(s) scheduled or no further sessions scheduled. When there were further sessions scheduled, the patients were sent: “Hi ___! We wanted to check in to see how your sessions are going so far! It looks like you have had ___ sessions and have ___ more scheduled which is great to see. We have been through NOCD Therapy previously, so we understand this process. Do you have any questions or concerns at this time?” When there were no further sessions scheduled the patients were sent: “Hello ___! We wanted to check in to see how your sessions are going so far! It looks like you have had ___ sessions with ___ and do not have any more scheduled at this time. We have been through NOCD therapy previously, so we understand this process. Do you have any questions or concerns at this time or anything that is preventing you from moving forward with your therapist or NOCD?” The messaging content was the same for everyone based on historical chart investigation and prior communication, when applicable. For example, if the pre-contact investigation showed a negative experience with the therapist, NOCD, or showcased a pending issue such as a billing problem the messaging was more specific. The final questions posed were: “Do you have any questions or concerns at this time?,” “Do you have any questions or concerns at this time or anything that is preventing you from moving forward with your therapist or NOCD?,” or “Do not hesitate to reach out if you need assistance!”

The second phase evolved when the patient replied, at which time the Member Advocate would respond to the message in a personalized manner based on chart investigation and prior communication. The Member Advocates’ goals were to build rapport and connection with these high-risk individuals and to elicit concerns or problems that may be affecting their therapeutic progress. Member Advocates then provided emotional support, assisted them with troubleshooting, and/or addressed concerns or problems that they were experiencing, with the intention of helping them adhere to treatment. The back-and-forth messaging could continue *ad lib* throughout treatment. Member Advocates did not specifically provide treatment or assist patients with treatment.

As mentioned, due to the limited availability of Member Advocates, they were not able to reach out to all of the high-risk individuals. Instead, *n* = 815 of *n* = 1,142 at risk were contacted. Retrospectively, this allowed for a naturalistic grouping that facilitated comparisons of adherence and OCD symptom reduction in those not at risk but not contacted, at-risk and contacted, at-risk and contacted but not engaged, and at-risk and contacted and engaged.

### Statistical analysis

Demographic variables and psychometric scores were compared among the three at-risk groups – not contacted, contacted but not engaged, and contacted and engaged – using ANOVA or Chi-squared tests. From this, we identified three variables that were significantly different among at-risk groups: gender, region, and DIAMOND severity. We then included these as covariates in the outcome analyses.

**Figure 1:**
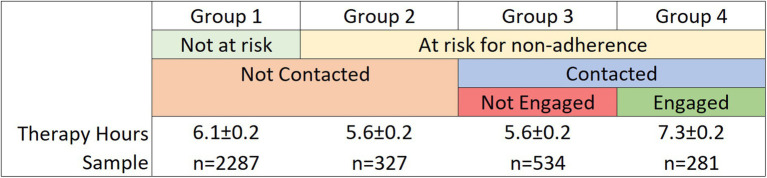
Mean therapy hours completed within 60 days and average dimensional obsessive-compulsive scale (DOCS) score change, by group type.

To evaluate outcomes, patients were grouped (retrospectively) as engaged or not engaged based on if they responded to peer support messages. The sample was divided into four groups: (1) not at-risk and not contacted, (2) at-risk and not contacted, (3) at-risk, contacted but not engaged, and (4) at-risk, contacted and engaged (Figure 1). For the primary outcome of adherence, we used a dimensional measure of hours of therapy completed within the first 60 days of treatment initiation. We chose this rather than a dichotomous measure of, for example, having reached at least 12 sessions, due to the fact that treatment benefits are not all-or-none with respect to number of sessions, and can vary across OCD patients in general. We compared the mean number of therapy hours completed among these groups using one-way ANOVA and follow-up independent sample *t*-tests. Multivariate linear regression was used to compare therapy hours completed (Supplementary Table S4) and, as a secondary outcome, changes in Dimensional Obsessive-Compulsive Scale (DOCS) (Abramowitz et al., 2010) scores (Supplementary Table S5). Outcomes were measured between baseline and the most recent assessment completed in 60 days, using the last observation carried forward. Statistical significance was determined using an alpha of 0.05, two-tailed, and outcomes were analyzed with R (R Foundation for Statistical Computing).

The analysis conducted in this study did not require research ethics board review as it does not meet the criteria for Human Subject Research as defined by federal regulations for human subject protections, 45 CFR 46.102(e); this is a secondary analysis of de-identified data from clinical records, obtained and analyzed retrospectively, and was not the result of a research intervention or interaction.

## Results

There were *N* = 3,429 patients included in the analysis (mean age = 29.3 ± 20.5 years) (Table 1).

**Table 1:** Baseline demographic and psychometric characteristics of the patient sample (*N* = 3,429).

Demographic characteristics	Valid *N*	Missing *N*	Percent	Mean	SD	Median
Age (years)^a^	3,426	3		28.02	11.35	26
Gender^b^	2,232	1,197				
Female	1,383		61.96			
Male	849		38.04			
Region	3,429	0				
Northeast USA	943		27.5			
Southeast USA	498		14.52			
Midwest USA	454		13.24			
Pacific USA	926		27			
Canada	45		1.31			
Other	563		16.42			
Payment method	3,429	0				
Insurance	2081		60.69			
Cash pay	1,348		39.31			
Baseline diagnostic scores^c^						
DOCS severity	2,867	562		26.27	13.76	25
DIAMOND severity	3,401	28		4.38	0.88	4
DASS depression	2,919	510		14.56	11.02	12
DASS anxiety	2,919	510		12.55	9.21	12
DASS stress	2,919	510		19.63	9.36	20
QLESQ-SF (Percentage)	2,500	929		54.23	16.32	54

a Age was self-reported, and invalid entries were counted as missing (<1 years and >120 years).

b Values for gender included ‘Male’, ‘Female’, or ‘Unknown’. In the data obtained from this sample, gender identification was an optional, self-reported field, and many chose not to answer.

c Diagnostic assessments were missing for some individuals, most often due to patients not completing them.

*N* = 1,142 were deemed at risk and *n* = 2,287 were deemed to not be at risk. As mentioned, due to the limited availability of Member Advocates, they were not able to reach out to all the high-risk individuals. Instead, *n* = 815 of *n* = 1,142 deemed at risk were contacted.

### Effect of engagement on therapy hours completed

Patients who were deemed not at-risk by the prediction model and not contacted by member advocates (“group 1”) completed a mean 6.1 ± 0.1 h of therapy within their first 60 days. Those who were at-risk but not contacted (“group 2”) completed a mean 5.6 ± 0.2 h. Of the patients who were contacted, those who did not engage (“group 3”) and those who did engage (“group 4”) completed a mean 5.6 ± 0.2 and 7.3 ± 0.2 h, respectively (See Supplementary Table S3 for the demographics and psychometrics for the subgroups).

**Figure 2:**
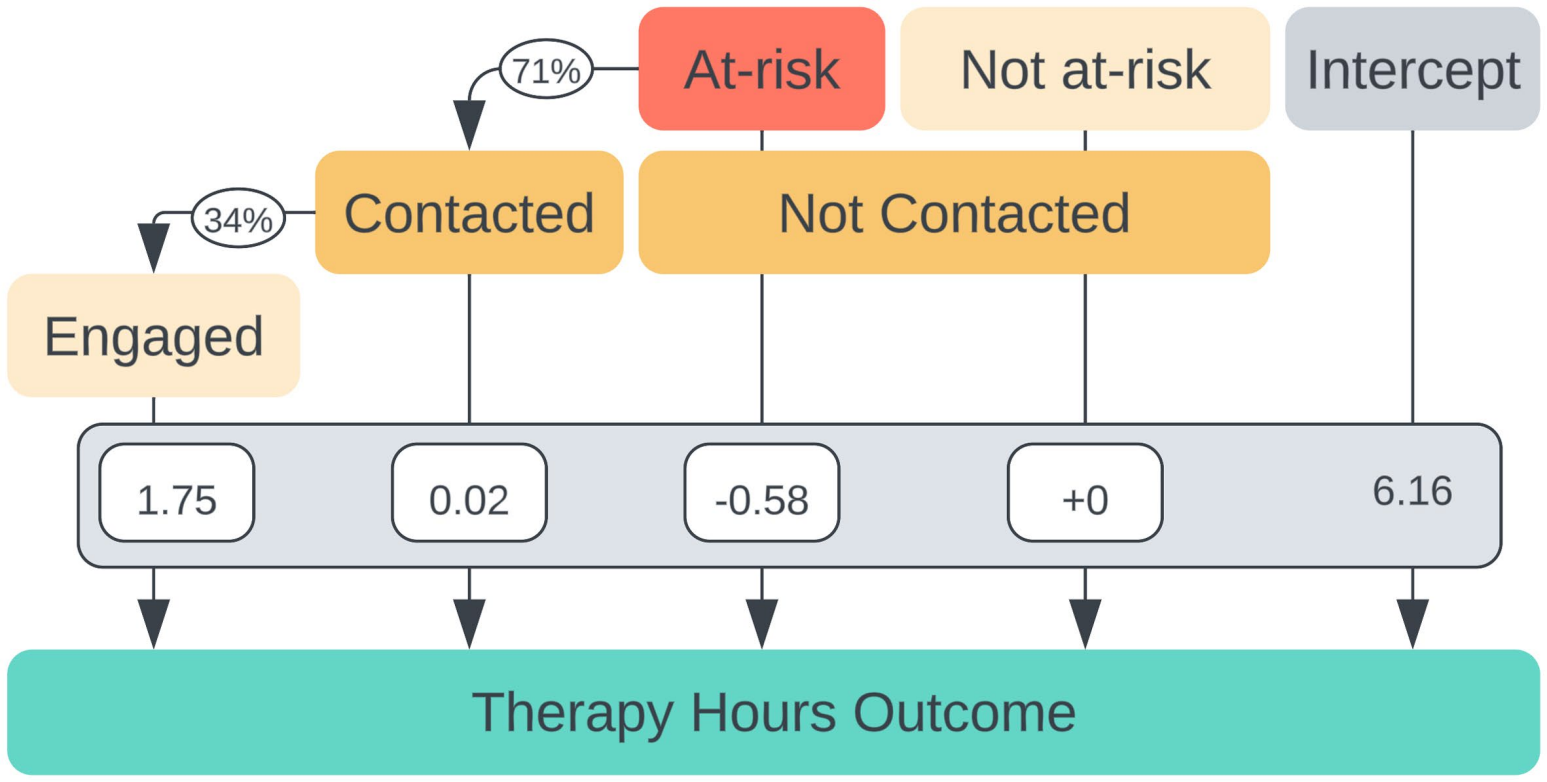
Multivariate regression coefficients for individual factors contributing to the number of therapy hours completed within 60 days of beginning treatment. The intercept represents a mean 6.16 therapy hours completed for individuals not at risk of non-adherence.

Compared to group 3, those who were contacted and engaged (“group 4,” *n* = 281) completed a statistically significant 1.75 more therapy hours within their first 60 days *F*(1, 3,440) = 30.85, *p* < 0.001 (Figure 2). The significant effect of engagement persisted when (as a post-hoc exploratory analysis) covariates of age and insurance versus cash pay were included. Insurance was a significant covariate (*p* < 0.001) affecting outcome; those with insurance coverage had a higher number of therapy hours.

An additional post-hoc *t*-test was performed to compare means of therapy hours completed in 60 days between the not at-risk, not contacted group (“group 1,” M = 6.1, SD = 0.2) and the at-risk, not contacted group (“group 2,” M = 5.6, SD = 0.2). Not-at-risk patients indeed had significantly greater hours of therapy completed than those who were identified as at-risk for non-adherence by the model, *t*(416.8) = 2.18, *p* = 0.030.

### Effects of engagement, being contacted, and being at risk on OCD symptom reduction

**Figure 3:**
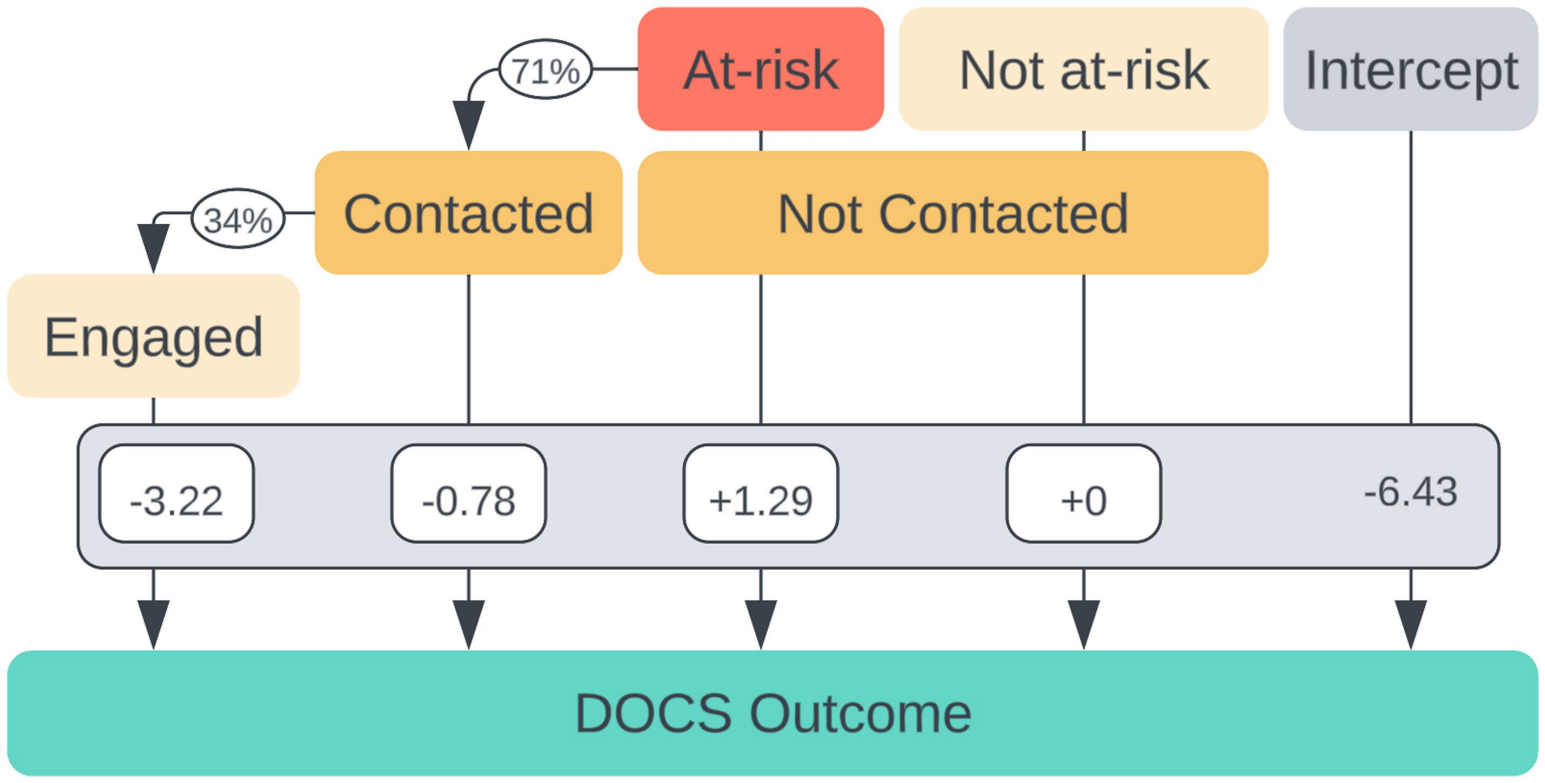
Multivariate regression coefficients for individual factors contributing to dimensional obsessive-compulsive scale (DOCS) score reduction additive to the baseline improvement of intercept = −6.43 ± 0.22.

In a linear regression model (Figure 3), having been engaged was associated with a significant 2.41 point reduction in DOCS outcomes *F*(1, 2,878) = 9.08, *p* = 0.004. The effect of being contacted itself was associated with a slight decrease in DOCS outcomes; however, the effect was not significant (*p* = 0.234, 95% CI −2.581, 0.631). The effect of being at risk was associated with a significant 1.53-point increase in DOCS score (*p* = 0.027).

There was a moderate linear relationship between time spent in therapy and DOCS scores for both the engaged group and the total patient population. The Pearson’s correlation coefficient (*r*) for the engaged group was −0.31 (df = 279, *p* < 0.001, 95% CI [−0.41, −0.21]), demonstrating that as time spent in therapy increased, DOCS scores decreased.

### Categories of messaging responses from patients

**Figure 4:**
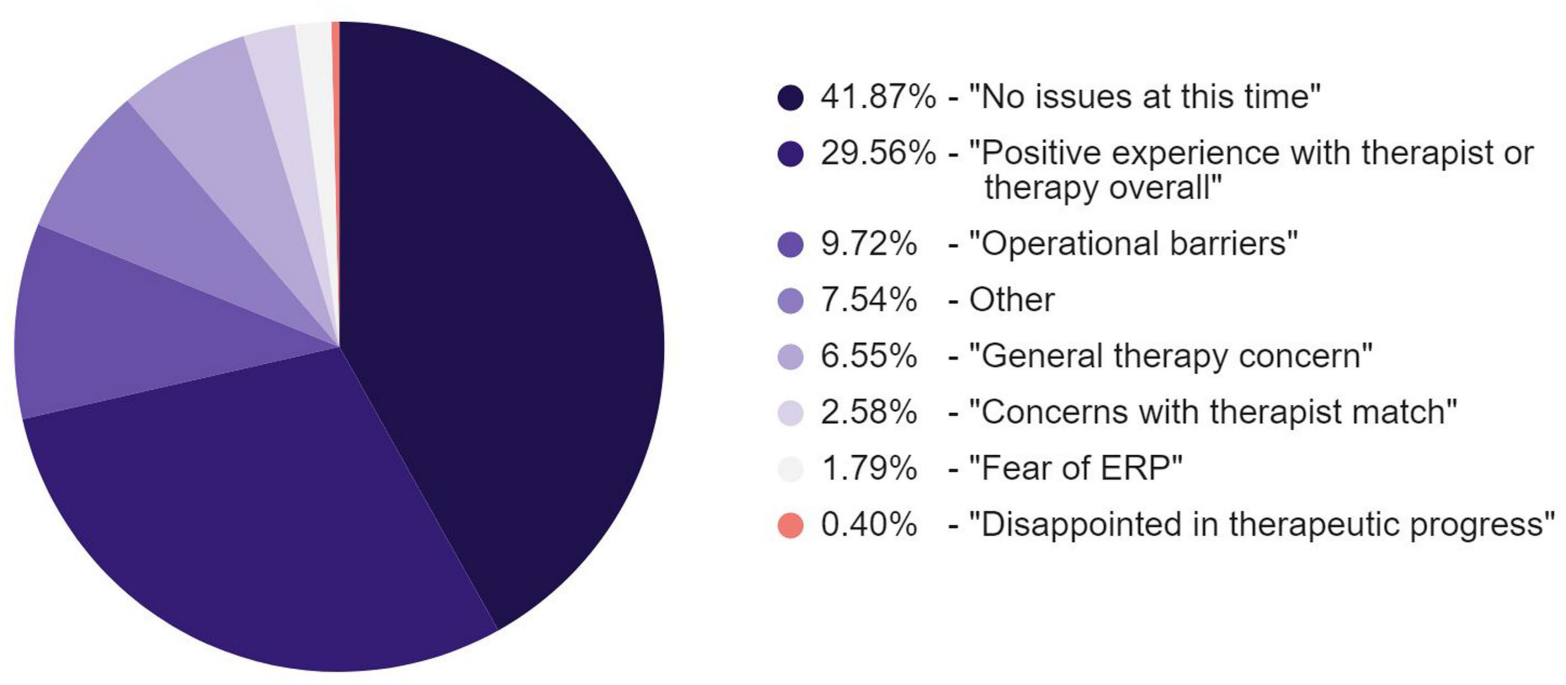
Response categories of engaged members to initial peer support messaging.

The responses from patients who engaged with peer support were manually labeled by the Member Advocates as falling into the following categories: no issues at this time, positive experience with therapist or therapy overall, operational barriers, general therapy concern, concerns with therapist match, fear of ERP, disappointment in therapeutic progress, and other (Figure 4).

Baseline demographic and psychometric characteristics of the patient sample are viewable in Table 1.

## Discussion

The results from this observational analysis show the effectiveness of a peer support intervention to improve adherence to ERP in those with OCD. This was facilitated by using a machine learning predictive algorithm to identify those at risk. For those at risk of non-adherence, those who engaged with peer advocacy had increased time spent in therapy within the first 60 days. This effect was significant and resulted in an average 1.75 more therapy hours completed (30.4% more) compared to those who were identified as at risk and contacted but did not engage. In addition, being engaged was associated with significantly greater OCD symptom reduction. Therefore, targeted peer advocate interventions may be an effective, cost-effective method for increasing therapy adherence for individuals with OCD and ultimately in reducing OCD symptoms.

Peer-delivered interventions have previously been demonstrated to improve self-reported outcomes in severe mental illnesses (Salyers et al., 2017) and medication adherence in persons with schizophrenia and other chronic mental illnesses (Finnerty et al., 2018). Yet, there remains a gap in understanding the distinct effects of peer support interventions on adherence to some mental illness treatments, including digital ERP. Patient attrition before peer intervention could be a contributor to this problem, as attrition in general is a challenge in mental health teletherapy (Fortuna et al., 2020). Our results illustrate the possibility of an effective targeted and tailored peer support intervention at critical, early time points in cognitive-behavioral therapy (ERP, specifically) for OCD that may reduce attrition. The benefits of peer–patient interactions, distinct from clinician–patient interactions, may come from “reciprocal accountability” in which both can mutually help each other and learn from each other (Fortuna et al., 2020). Also, within these relationships can be autonomy, shared lived experience (importantly, including ERP treatment), bonding, and providing a sense of hope.

In pursuit of scaling telehealth options to larger populations, it may be important to efficiently select and intervene with patients at high risk of poor clinical outcomes. Peer support intervention is associated with cost, in addition to there being a limited pool of individuals available and interested in being in this role. Thus, to optimize efficiency, the identification of at-risk patients could allow for cost-effective patient selection for targeted interventions.

Further, although digital messaging is a relatively low-cost intervention, it should not necessarily be distributed to all patients since some may not need or want peer support assistance. Patients may be annoyed or frustrated receiving non-specific “blasts” of messages, particularly when they are not relevant or needed for their unique situation. Because of limited resources, it may not be feasible to send initial personalized messages to all patients since it requires research into patients’ individual situations. Also, it may not be efficient to do so, given the low response rate of patients. On the other hand, using only generic messages runs the risk of it feeling impersonal since they may not be relevant to their situations. Further, even those at risk may not be comfortable, want, or need engagement with peer support; this was evidenced in this analysis in which only 34.5% of those predicted to be at risk engaged with a Member Advocate after being contacted. Selective digital messaging, therefore, narrows the pool of people who are both in need of, and willing to engage with, peer support.

A limitation of this analysis is that it is retrospective and observational, rather than a controlled clinical trial with factors controlled through, e.g., randomization and with a control intervention. The causal nature of the interaction that appeared to be effective (engagement with the member advocate, rather than just being contacted) cannot definitively be established since it is possible that those who were more likely to engage were also those who were more likely to be adherent with treatment and have better symptom improvements. Another limitation of the effectiveness of these procedures could have stemmed from the modest accuracy of our prediction model, having an AUC of only 0.653. This was the most accurate model compared with another linear model, support vector classification (Cortes and Vapnik, 1995), and random forest classifier, a nonlinear model (Kulkarni et al., 2016). It can be challenging to obtain high accuracy for predicting an outcome that may be the result of complex environmental, situational, and psychological/psychiatric factors (including individual patient factors, therapist factors, and dynamic patient-therapist factors). Further adding to this challenge is the heterogeneity of the population itself, encompassing a wide range of ages from 4 to 88, different subtypes of OCD, some having one or more comorbidities, being medicated or unmedicated, having done ERP previously, etc. The childrens’ parents or guardians were contacted by Member Advocates while for the adolescents either they or their parents or guardians were contacted; however, data were analyzed and interpreted uniformly as this distinction was not annotated. In addition, we had incomplete gender data. Nearly half of the overall sample did not provide gender information, as it was optional for patients to enter when registering for treatment. Thus, the logistic regression prediction model was not trained with gender as a feature; this may or may not have been an important contributor to the multivariate prediction model. Quantitative data regarding the frequency and duration of engagement with Member Advocates was not specifically recorded due to the high volume and mixture of messages related to other topics. In future works, this could be valuable to characterize peer support interventions. Despite the low accuracy, these results suggest that the implementation of this prediction model for identifying and subsequently engaging and intervening with those at high risk for non-adherence, compared with those at high risk who were not engaged in the intervention, resulted in meaningful clinical outcomes of increasing total hours spent in therapy and additional symptom improvements. Future models, as sample sizes permit, might be tested for subgroups (e.g., by age or symptom subtype), which may prove to be more accurate.

## Conclusion

Providing individuals with OCD with a peer support intervention may result in beneficial clinical outcomes for those who are at high risk of non-adherence to video ERP teletherapy. In large patient populations, leveraging machine learning methods for targeted interventions may be a useful and efficient clinical strategy. In doing so, health and human resources can be better allocated to patients who can benefit most from individual, personalized contact. Future models that have improved prediction accuracy for high-risk patients, e.g., those that could be developed and applied to subgroups, might result in improved patient targeting and thereby increase the overall efficiency of peer support interventions.

## Data availability statement

The raw data supporting the conclusions of this article will be made available by the authors, without undue reservation.

## Ethics statement

Ethical approval was not required for the study involving humans in accordance with the local legislation and institutional requirements. Written informed consent to participate in this study was not required from the participants or the participants’ legal guardians/next of kin in accordance with the national legislation and the institutional requirements.

## Author contributions

CM wrote the paper, contributed to the analyses and data collection, interpreted results, and finalized the submission of the manuscript. AR wrote the paper, interpreted results, designed, performed the statistical analyses, and finalized the submission of the manuscript. JK interpreted the results and wrote the paper. SA and JH designed and performed logistic regression prediction modeling. KO and KL assisted with writing the paper. RM, LT, and SS aided with interpreting results. JF wrote the paper, designed the analyses, and finalized the submission of the manuscript. All authors contributed to the article and approved the submitted version.

## Abbreviations

DOCS: dimensional obsessive-compulsive scale (Abramowitz et al., 2010)
DIAMOND: diagnostic interview for anxiety, mood, and OCD and related neuropsychiatric disorders (Tolin et al., 2018)
DASS: depression, anxiety, and stress scales (21-item) (Lovibond and Lovibond, 1995)
QLES-Q: quality of life enjoyment and satisfaction questionnaire – short form (Endicott et al., 1993)
