# Supplementary material for: A targeted strategic peer support intervention to increase adherence to video teletherapy exposure and response prevention treatment for obsessive-compulsive disorder: a retrospective observational analysis
Source: Front Hum Neurosci. 2023 Oct 30;17:1251194. doi: 10.3389/fnhum.2023.1251194 (PMC10643166; doi:10.3389/fnhum.2023.1251194)
Supplement: Supplementary file 1 [file Data_Sheet_1.docx]

Supplementary Material

A targeted strategic peer support intervention to increase adherence to video teletherapy exposure and response prevention treatment for obsessive-compulsive disorder: a retrospective observational analysis

Christopher E. Murphy, Andreas Rhode, Jeremy Kreyling, Scott Appel, Jonathan Heintz, Kerry Osborn, Kyle Lucas, Reza Mohideen, Larry Trusky, Stephen Smith, Jamie D. Feusner^*^

***Correspondence:** Corresponding Author: Jamie@ nocdhelp.com

# Supplementary Tables

Table S1. Baseline demographic and psychometric characteristics of the logistic regression model train/test sample (N=13809).

| **Demographic Characteristics** | **Valid *N*** | **Missing *N*** | **Percent** | **Mean** | **SD** | **Median** |
| --- | --- | --- | --- | --- | --- | --- |
| Age (years)ᵃ | 13809 |  |  | 27.96 | 10.66 | 26 |
| Region | 13809 |  |  |  |  |  |
| Northeast USA | 3921 |  | 28.39 |  |  |  |
| Southeast USA | 1997 |  | 14.46 |  |  |  |
| Midwest USA | 2044 |  | 14.80 |  |  |  |
| Pacific USA | 3236 |  | 23.43 |  |  |  |
| Canada | 198 |  | 1.43 |  |  |  |
| Other | 2413 |  | 17.47 |  |  |  |
| Payment Method | 13809 |  |  |  |  |  |
| Insurance | 7115 |  | 51.52 |  |  |  |
| Cash Pay | 6694 |  | 48.48 |  |  |  |
| **Baseline Diagnostic Scores^b^** |  |  |  |  |  |  |
| DOCS Severity | 13809 |  |  | 25.94 | 12.87 | 24 |
| DIAMOND Severity | 13809 |  |  | 4.49 | 0.87 | 4 |
| DASS Depression | 13809 |  |  | 14.62 | 10.66 | 12 |
| DASS Anxiety | 13809 |  |  | 12.07 | 8.62 | 10 |
| DASS Stress | 13809 |  |  | 19.30 | 9.01 | 18 |
| QLESQ-SF (Percentile) | 13809 |  |  | 55.46 | 15.73 | 55.63 |

ᵃAge was self-reported, and invalid entries were counted as missing (<1 years and >120 years)

^b^Diagnostic assessments were missing for some individuals, most often due to patients not completing them.

Table S2. List of features and associated odds ratios of being adherent used in training the logistic regression prediction model.

| **Feature Name** | **Odds Ratio** | **Lower 95%** | **Upper 95%** | **Prob** |
| --- | --- | --- | --- | --- |
| Therapist's star rating: 4/5 v 1/5 | 2.004 | 1.62845 | 2.46613 | <.0001 |
| DIAMOND distress score group: 5 v 1-2 | 1.92321 | 1.35394 | 2.73183 | 0.0003 |
| DIAMOND distress score group: 6+ v 1-2 | 1.77881 | 1.22615 | 2.58054 | 0.0024 |
| DIAMOND distress score group: 4 v 1-2 | 1.72578 | 1.22018 | 2.44088 | 0.002 |
| DIAMOND distress score group 3: v 1-2 | 1.53803 | 1.08525 | 2.17971 | 0.0155 |
| DOCS severity score group: 50+ v 0-15 | 1.5063 | 1.22654 | 1.84986 | <.0001 |
| Number of mobile app opens during treatment period: 51+ v 0 | 1.46984 | 1.20187 | 1.79754 | 0.0002 |
| Comorbidity status: YES v No | 1.38973 | 1.07234 | 1.80105 | 0.0128 |
| Therapist's star rating: 3/5 v 1/5 | 1.36889 | 1.17467 | 1.5952 | <.0001 |
| Billing plan at baseline: Insurance v Cash Pay | 1.34993 | 1.25322 | 1.4541 | <.0001 |
| Therapist's licensing level: PhD v Masters | 1.32003 | 1.11903 | 1.55714 | 0.001 |
| DOCS severity score group: 30-50 v 0-15 | 1.23306 | 1.10215 | 1.37951 | 0.0003 |
| SMS messages from patients: 10+ vs 0-1 | 1.2056 | 1.03126 | 1.40942 | 0.019 |
| DOCS severity score group: 15-30 v 0-15 | 1.19021 | 1.0782 | 1.31386 | 0.0006 |
| SMS messages from clinicians: 8-12 vs 0 | 1.18916 | 1.00537 | 1.40654 | 0.0431 |
| SMS messages from patients: 5-9 vs 0-1 | 1.16818 | 1.01347 | 1.34652 | 0.032 |
| Current medication status: Unknown vs. No | 1.1454 | 0.87752 | 1.49504 | 0.3179 |
| Number of mobile app opens during treatment period: 13-50 v 0 | 1.14232 | 1.02157 | 1.27734 | 0.0196 |
| SMS messages from clinicians: 13-19 vs 0 | 1.13499 | 0.95071 | 1.355 | 0.1613 |
| SMS messages from clinicians: 19+ vs 0 | 1.11591 | 0.92588 | 1.34495 | 0.2496 |
| SMS messages from patients: 2-4 vs 0-1 | 1.09756 | 0.95875 | 1.25649 | 0.1773 |
| Therapist's star rating: 2 v 1 | 1.09418 | 0.98377 | 1.21696 | 0.0972 |
| Current medication status: Yes vs. No | 1.06937 | 0.96525 | 1.18474 | 0.1994 |
| SMS messages from clinicians: 1-7 vs 0 | 1.05845 | 0.89608 | 1.25025 | 0.5038 |
| Number of discussion posts written during treatment period: 1-4 v 0 | 1.04348 | 0.85841 | 1.26846 | 0.6692 |
| Baseline DASS stress assessment score* | 1.03174 | 1.01503 | 1.04872 | 0.0002 |
| REGION Other Country v Northeast | 1.0295 | 0.73087 | 1.45017 | 0.8679 |
| Baseline QLES assessment score* | 1.00974 | 0.997 | 1.02264 | 0.1343 |
| Number of mobile app opens during treatment period: 1-12 v 0 | 1.00834 | 0.92149 | 1.10338 | 0.8565 |
| Baseline QLES assessment score squared* | 0.99993 | 0.99982 | 1.00005 | 0.2414 |
| Baseline DASS stress assessment score squared* | 0.99934 | 0.99896 | 0.99973 | 0.0008 |
| Baseline DASS depression assessment score* | 0.99187 | 0.9869 | 0.99687 | 0.0015 |
| DIAMOND functional score group: 3 v 1-2 | 0.93252 | 0.76644 | 1.13459 | 0.4849 |
| Patient region of residence: Pacific v Northeast | 0.93021 | 0.8414 | 1.02841 | 0.1577 |
| DIAMOND functional score group: 4 v 1-2 | 0.90385 | 0.74077 | 1.10282 | 0.3192 |
| DIAMOND functional score group: 5 v 1-2 | 0.89804 | 0.72554 | 1.11154 | 0.323 |
| Pre-therapy intake no-shows: 3+ v 0 | 0.84675 | 0.39361 | 1.82157 | 0.6704 |
| Pre-therapy intake no-shows: 2 v 0 | 0.80911 | 0.52248 | 1.25297 | 0.3425 |
| Pre-therapy intake no-shows: 1 v 0 | 0.78699 | 0.67424 | 0.91859 | 0.0024 |
| DIAMOND functional score group: 6+ v 1-2 | 0.7718 | 0.59051 | 1.00875 | 0.0579 |
| Number of discussion posts written during treatment period: 5+ v 0 | 0.75262 | 0.59183 | 0.9571 | 0.0205 |
| Patient region of residence: Canada v Northeast USA | 0.75249 | 0.6498 | 0.87141 | 0.0001 |
| Patient region of residence: Southeast USA v Northeast USA | 0.74429 | 0.66515 | 0.83284 | <.0001 |
| Patient region of residence: Midwest USA v Northeast USA | 0.74072 | 0.66131 | 0.82968 | <.0001 |
| Patient region of residence: South/West USA v Northeast USA | 0.72489 | 0.6376 | 0.82412 | <.0001 |
| First therapy appointment cancellations: Yes v No | 0.69222 | 0.63164 | 0.7586 | <.0001 |
| Completed patient intake: Yes v No | 0.62836 | 0.52851 | 0.74708 | <.0001 |
| Second therapy appointment cancellations: Yes v No | 0.59779 | 0.53765 | 0.66466 | <.0001 |
| Third therapy appointment cancellations: Yes v No | 0.47182 | 0.42505 | 0.52375 | <.0001 |
| Intercept | 0.44198 | 0.24045 | 0.81243 | 0.0086 |

*Note*. Features were binarized and the corresponding levels are listed following the feature name (e.g., “Feature name: Level 1 v Level 2”)

Table S3. Demographic characteristics of the four study subgroups: Group 1, not at-risk for non-adherence and not contacted or engaged; Group 2, at-risk for non-adherence, but not contacted or engaged; Group 3, at-risk for non-adherence and contacted but not engaged; and Group 4, at-risk for non-adherence, contacted and engaged. One-way ANOVA and chi-square tests were used to compare differences in means among at-risk groups (Groups 2-4). ‘Gender’, ‘Region’, and ‘DIAMOND Severity’ significantly differed among groups.

|  | **Group 1** | **Group 2** | **Group 3** | **Group 4** | ***p*-value** | **Statistic** |
| --- | --- | --- | --- | --- | --- | --- |
| **Ageᵃ** (years) |  |  |  |  | 0.200 | *F* = 1.610 |
| Valid N (%) | 2287 (100) | 322 (98.47) | 534 (100) | 281 (100) |  |  |
| Mean (SD) | 28.16 (11.35) | 27.27 (11.39) | 27.44 (11.05) | 28.77 (11.86) |  |  |
| Median | 26 | 25 | 25 | 27 |  |  |
| Range | 4, 88 | 5, 75 | 7, 29 | 5, 75 |  |  |
| **Genderᵇ**, n (%) |  |  |  |  | 0.022 | *X^2^* = 7.616 |
| Female | 1032 (45.12) | 111 (34.37) | 154 (28.84) | 85 (30.25) |  |  |
| Male | 573 (25.05) | 61 (18.89) | 145 (27.15) | 70 (24.91) |  |  |
| Unknown | 682 (29.82) | 151 (46.75) | 235 (44.01) | 126 (44.84) |  |  |
| **Region,** n (%) |  |  |  |  | 0.039 | *X^2^* = 19.130 |
| Northeast USA | 804 (35.16) | 35 (10.84) | 53 (9.93) | 50 (17.79) |  |  |
| Southeast USA | 233 (10.19) | 75 (23.22) | 121 (22.66) | 67 (23.84) |  |  |
| Midwest USA | 186 (8.13) | 83 (25.70) | 123 (23.03) | 62 (22.06) |  |  |
| Pacific USA | 640 (27.98) | 77 (23.84) | 151 (28.28) | 57 (20.28) |  |  |
| Canada | 28 (1.22) | 3 (0.93) | 7 (1.31) | 7 (2.49) |  |  |
| Other | 396 (17.32) | 50 (15.48) | 79 (14.79) | 38 (13.52) |  |  |
| **Payment Method,** n (%) |  |  |  |  | 0.789 | *X^2^* = 0.475 |
| Insurance | 1532 (66.99) | 150 (46.44) | 258 (48.31) | 138 (49.11) |  |  |
| Cash Pay | 755 (33.01) | 173 (53.56) | 276 (51.69) | 143 (50.89) |  |  |
| **DOCS Severity** |  |  |  |  | 0.746 | *F* = 0.294 |
| Valid N (%) | 1093 (47.79) | 185 (56.57) | 253 (47.39) | 195 (69.40) |  |  |
| Mean (SD) | 26.66 (14.30) | 26.78 (12.89) | 27.64 (13.00) | 27.64 (12.59) |  |  |
| Median | 24 | 26 | 27 | 25 |  |  |
| Range | 1, 77 | 1, 77 | 1, 75 | 1, 67 |  |  |
| **DIAMOND Severity** |  |  |  |  | 0.024 | *F* = 3.45 |
| Valid N (%) | 2215 (96.85) | 318 (97.25) | 515 (96.44) | 268 (95.37) |  |  |
| Mean (SD) | 4.37 (0.87) | 4.64 (0.94) | 4.48 (0.85) | 4.52 |  |  |
| Median | 4 | 5 | 4 | 4 |  |  |
| Range | 1, 7 | 1, 7 | 1, 7 | 2, 7 |  |  |
| **DASS Depression** |  |  |  |  | 0.147 | *F* = 1.922 |
| Valid N (%) | 1159 (50.68) | 190 (58.10) | 269 (50,37) | 205 (72.95) |  |  |
| Mean (SD) | 11.34 (9.64) | 21.38 (11.57) | 20.07 (11.92) | 19.03 (12.14) |  |  |
| Median | 10 | 22 | 22 | 18 |  |  |
| Range | 0, 42 | 0, 42 | 0, 42 | 0, 42 |  |  |
| **DASS Anxiety** |  |  |  |  | 0.197 | *F* = 1.628 |
| Valid N (%) | 1159 (50.68) | 190 (58.10) | 269 (50.37) | 205 (72.95) |  |  |
| Mean (SD) | 11.34 (8.35) | 17.87 (9.85) | 16.50 (10.19) | 16.15 (10.15) |  |  |
| Median | 10 | 18 | 16 | 14 |  |  |
| Range | 0, 42 | 0, 42 | 0, 40 | 0, 38 |  |  |
| **DASS Stress** |  |  |  |  | 0.181 | *F* = 1.713 |
| Valid N (%) | 1159 (50.68) | 190 (58.10) | 269 (50.37) | 205 (72.95) |  |  |
| Mean (SD) | 18.57 (8.61) | 24.11 (8.82) | 22.51 (9.33) | 22.91 (9.48) |  |  |
| Median | 18 | 24 | 22 | 24 |  |  |
| Range | 0, 42 | 2, 42 | 0, 42 | 0, 42 |  |  |
| **QLESQ-SF** (Percentile) |  |  |  |  | 0.164 | *F* = 1.815 |
| Valid N (%) | 999 (43.68) | 157 (48.01) | 233 (43.63) | 181 (64.41) |  |  |
| Mean (SD) | 57.02 (14.49) | 45.06 (15.58) | 45.90 (17.58) | 48.41 (18.01) |  |  |
| Median | 58 | 45 | 45 | 46 |  |  |
| Range | 7, 100 | 11, 88 | 2, 100 | 10, 93 |  |  |

Table S4. Complete multivariate regression coefficients for the primary outcome, therapy hours completed in 60 days. A full model with controls for significant covariates did not change the direct effect of treatment.

| **Parameter** | **Coefficient** | **SE** | **95% CI** | ***t*** | ***p-*value** |
| --- | --- | --- | --- | --- | --- |
| (Intercept) | 4.58 | 0.34 | [3.91, 5.26] | 13.37 | <.001 |
| Engaged | 1.68 | 0.28 | [1.13, 2.23] | 5.96 | <.001 |
| Contacted | 0.11 | 0.26 | [-0.40, 0.63] | 0.42 | 0.671 |
| At-risk | -0.46 | 0.23 | [-0.91, -0.01] | -2.01 | 0.044 |
| Billing plan-Cash pay | -1.22 | 0.14 | [-1.49, -0.95] | -8.92 | <.001 |
| Region-Midwest | 0.90 | 0.20 | [0.51, 1.30] | 4.47 | <.001 |
| Region-Northeast | 0.59 | 0.15 | [0.29, 0.90] | 3.82 | <.001 |
| DIAMOND Severity | 0.39 | 0.08 | [0.25, 0.54] | 5.25 | <.001 |

Table S5. Complete multivariate regression coefficients for the secondary outcome, change between baseline and last DOCS scores recorded in 60 days. A full model with controls for significant covariates did not change the direct effect of treatment.

| **Parameter** | **Coefficient** | **SE** | **95% CI** | ***t*** | ***p-*value** |
| --- | --- | --- | --- | --- | --- |
| (Intercept) | -4.75 | 0.90 | [-6.52, -2.98] | -5.26 | <.001 |
| Engaged | -3.24 | 0.76 | [-4.73, -1.76] | -4.28 | <.001 |
| Contacted | -0.86 | 0.71 | [-2.25, 0.52] | -1.22 | 0.221 |
| At-risk | 0.88 | 0.61 | [-0.31, 2.07] | 1.46 | 0.145 |
| Billing plan-Cash pay | 2.69 | 0.37 | [1.97, 3.41] | 7.33 | <.001 |
| DIAMOND Severity | -0.58 | 0.20 | [-0.98, -0.19] | -2.88 | 0.004 |
